# A UK-based ground truth data set of GCMS analysed ignitable liquid samples — a template for making chromatographic data accessible as an open source data set.

**DOI:** 10.1016/j.dib.2022.108670

**Published:** 2022-10-13

**Authors:** Jonathan Miller, Roberto Puch-Solis, Wan Nur Syuhaila Mat Desa, Niamh Nic Daeid

**Affiliations:** a Leverhulme Research Centre for Forensic Science, School of Science and Engineering, University of Dundee, Nethergate, Dundee DD2 1HD, Scotland; b Forensic Science Programme, School of Health Sciences, Health Campus, Universiti Sains Malaysia, Kubang Kerian 16150, Kelantan, Malaysia

**Keywords:** Ignitable Liquids, GCMS, Statistical modelling data, Machine learning data

## Abstract

Fire debris is often recovered as part of a fire scene investigation to determine whether an ignitable liquid might be present which may be evidence of a deliberate fire. The analysis of fire debris produces chromatograms that a forensic chemist uses to determine whether or not an ignitable liquid may be present. Currently there are very few publicly available data sets that can be used for training and statistical modelling in this area. The data set in this paper has been prepared with these two applications in mind and covers a wide range of ignitable liquids available in the UK. We created a data set of 35 ignitable liquids including petrol (gasoline), light, medium and heavy petroleum distillates (i.e diesel) from several retailers. Each ignitable liquid was systematically evaporated to produce six additional samples. Each sample was repetitively analysed to provide an overall data set of 751 analytical outputs (including chromatograms). Each data sample is expressed in multiple formats and the metadata containing any data used in the production of the samples is included. The folder and file names are designed to avoid misplacements and to manipulate folders and files systematically using computer code.

**Specification Table**

**Table:** 

Subject	Analytical Chemistry
Specific subject area	Collection of off-the-shelf ignitable liquids (ILs) from local Scottish businesses to study the interpretation of IL patterns in fire debris analysis
Type of data	Binary (Raw) Table Graph Report
How data were acquired	Data acquisition occurred in three distinct phases. Phase I consisted of the in-person collection of ILs from Scottish businesses. In phase II the data was processed and analysed using gas chromatography mass spectrometry (GCMS) from Agilent Systems using Hewlett-Packard (HP) 6890/5973 MS ChemStation (version B.00.01 Hewlet Packard, Agilent technologies), following ASTM E1618 protocols. In Phase III the data was engineered using Agilent’s proprietary software and open source software: OpenChrom 1.4 [1], Julia 1.7 [2] and Linux (Ubuntu 21.04) [3].
Data format	Raw Analysed Filtered
Parameters for data collection	GCMS standard operating procedures were adhered to for sample analysis, including temperature, run times, carrier gas and internal standards. ILs were grouped into classifications (e.g. diesel), sub-classifications (e.g. regular), brands (e.g. Asda), weathering (degree of evaporation), and sample replicate number.
Description of data collection	ILs were purchased and collected from local Scottish retailers. All ILs were stored in pre-sealed or clean fuel containers and stored at room temperature in darkness. ILs were systematically evaporated to predetermined amounts. Mass spectrometry and chromatography information was extracted via standard GCMS techniques. Outputted data was programmatically engineered into accessible formats using open source software.
Data source location	Institution: University of Dundee City: Dundee Country: Scotland, UK
Data accessibility	Repository name: Discovery research portal - https://discovery.dundee.ac.uk/[4] Data identification number: https://doi.org/10.15132/10000178 Direct URL to data: https://doi.org/10.15132/10000178 Instructions for accessing these data: The dataset (3.3 GB) is stored online as a ZIP file.
Related research article	W.N.S. Mat-Desa, D. Ismail, N. NicDaeid, Classification and Source Determination of Medium Petroleum Distillates by Chemometric and Artificial Neural Networks: A Self Organizing Feature Approach, Analytical chemistry 83 (2011) 7745-7754. https://doi.org/10.1021/ac202315y[5].

## Value of the Data

• The identification of ILs, if present at a fire scene, has the potential to provide corroborative support for deliberate fire setting. The chemical composition of ILs and how these change as they are exposed to heat is a very informative and important component of understanding the composition and possible classification of an IL. The data set is a collection of a diverse range of commonly encountered ILs which have been systematically evaporated to create a bespoke ground truth data set to contribute to the interpretation of fire debris.
• The data set can be used in machine learning and artificial intelligence algorithms for IL classification. It can also provide a building block for the development of prediction algorithms to triage fire debris samples, enabling practitioners to concentrate only on samples of relevance, thereby improving efficiency and work flow. In addition, the data set can be used for training of forensic scientists.
• The data engineering and folder/file name structure provides a framework for production and sharing IL data to underpin wider knowledge for practice and research. The framework can be used to create a comprehensive, shareable and robust data set of other ILs that can be used in conjunction with this data sets to form a large collection of data.

## 1 Data Description

Deliberate fires may be started and accelerated using an IL. An IL is defined as “any liquid or the liquid phase of any material that is capable of fuelling a fire, including a flammable liquid, combustible liquid, or any other material that can be liquefied and burnedǥ [6]. The chemical compounds that compose an IL delineates its category which can be petroleum or non-petroleum based. Records show that petroleum based ILs are the most common form recovered from deliberate fires [7]. The data set consists of petroleum based ILs only, classified as petrol or a petroleum distillate product (although other classifications may also be used on the data if desired). Specifically,

1. Petrol
2. Light Petroleum Distillate (LPD)
3. Medium Petroleum Distillate (MPD)
4. Heavy Petroleum Distillate (Diesel)

**Fig. 1:**
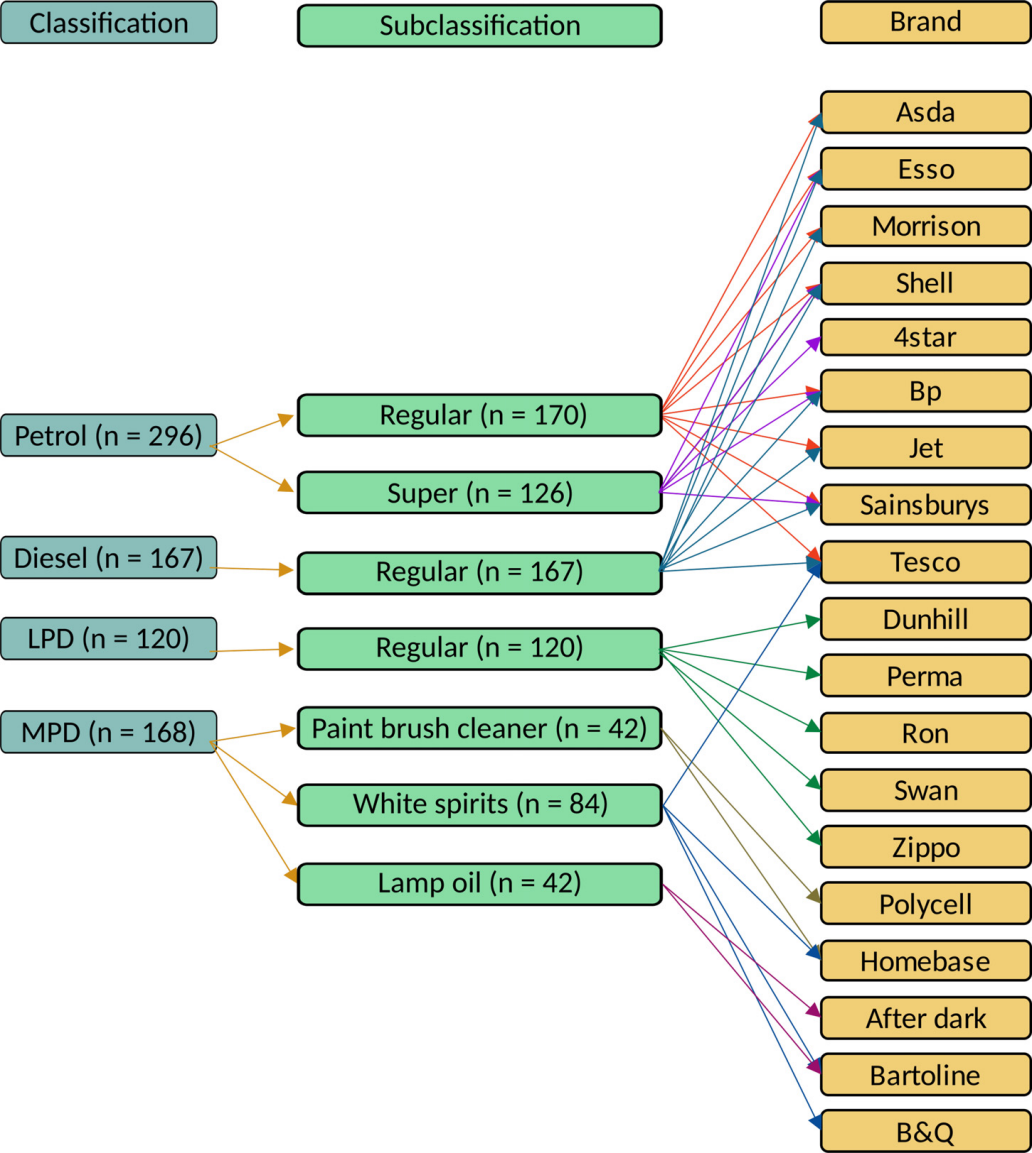
Classification of ILs.

The types of ILs and the retailers where they were purchased from are shown in Fig. 1 and the specific number of samples from each retailer are given in Table 1.

**Table 1:** Count of each brand within a classification.

Classification	Brand	Count
LPD	Swan	21
	Perma	21
	Zippo	36
	Ronsonol	21
	Dunhill	21

MPD	Homebase	42
	B&Q	21
	Bartoline	42
	After-Dark	21
	Tesco	21
	Polycell	21

Petrol	Morrison	20
	Jet	21
	Asda	21
	Shell	42
	Tesco	42
	BP	45
	Sainsbury	42
	Esso	42
	Leaded-4-Star	21

Diesel	Morrison	20
	Jet	21
	Asda	21
	Shell	21
	Tesco	21
	BP	21
	Sainsbury	21
	Esso	21

A set of partially evaporated samples for each IL at approximately 10, 25, 50, 75, 90 and 95 percent evaporation were prepared. Prior to instrumental analyses, each sample (unevaporated and evaporated) was diluted to 2% in pentane with 0.5 mg/mL tetrachloroethylene as internal standard (ISTD). Each evaporated sample, together with an unevaporated sample of the IL, was analysed using GCMS.

**Fig. 2:**
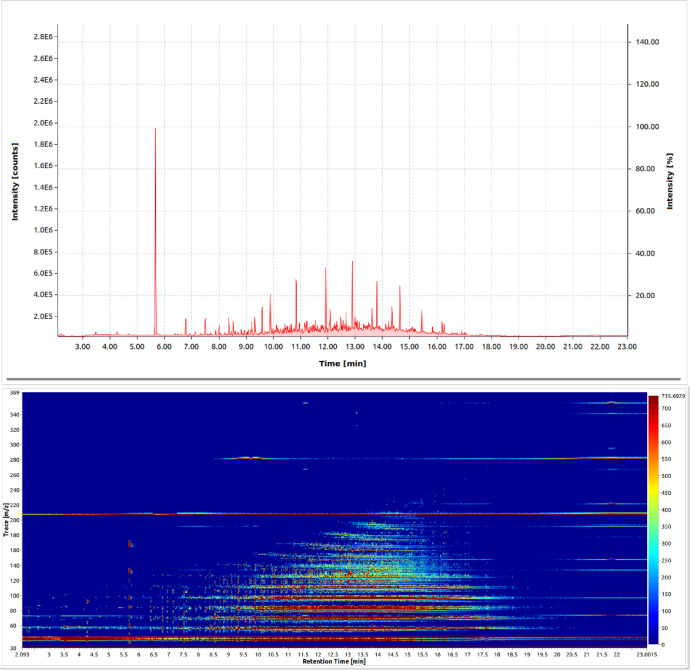
A chromatogram (top) and a heatmap (bottom) of a sample of regular diesel from Asda. This sample also contained the ISTD and pentane peak.

A chromatogram and a heat map were produced for each sample, Fig. 2. As the samples contain a pentane and an ISTD peak, four sets of heat maps were produced for each sample, with (a) both the pentane and ISTD peaks present, either (b) the pentane or (c) the ISTD peaks present, and (d) neither the pentane nor the ISTD peaks present.

The data set was organised into folders where each folder contained a single sample. Each sample folder was assigned a unique name which contained the defining information of the sample. This prevents data from being allocated to the wrong place while enabling management of the data programmatically. An example of a sample folder name is:

ILweathered_Diesel_Regular_Asda_weather-00_sample-1_30080926.D, where,

1. “ILweathered”. Prefix that is the same for all sample folders that identifies the data set as ILs that have been evaporated.
2. “Diesel”. This is the classification of the IL. Possible values are: “Petrol”, “LPD”, “MPD” and “Diesel”.
3. “Regular”. This is the sub-classification of the IL. Possible values are: “Regular”, “Super”, “Lamp-Oil”, “Paint-Brush-Cleaner” and “White-Spirits”.
4. “Asda”. This is the brand of the IL. Possible values are “Swan”, “Homebase”, “Morrison”, “B&Q”, “Jet”, “Perma”, “Asda”, “Bartoline”, “After-Dark”, “Shell”, “Tesco”, “Zippo”, “Ronsonol”, “BP”, “Sainsbury”, “Esso”, “Dunhill”, “Polycell”, “Leaded-4-Star”.
5. “weather-00”. This part is the weathered amount of the IL. Possible values which can appear in addition to “00” (which is the unevaporated sample) are, “10”, “25”, “50”, “75”, “90” and “95” which relate to the percentage of weathering that the sample has undergone.
6. “sample-1”. The number “1” refer to replicate number. Possible values are 1−24.
7. “30080926.D” This part is the Agilent identifier of the IL. The Agilent Chemstation used the date of creation and iteration number of sample analysed to compose a unique identifier.

Each sample folder contains raw data, figures, tables and reports. The folder name storing all of the files associated with the same sample should be the same as the sample name. The name of each file within the folder, to protect data integrity, should also contain the sample name with the exception of any raw data files produced by instrument software. These files are required to be readable by OpenChrom in order that the analytical output can be accessed independently of the GCMS instrument. In the data presented, the GCMS instrument software was Agilent Chemstation software which produces the files “ANALYTICALMETHODS_IGNITABLE_LIQUIDS.M”, “DATA.MS”, “GC01A.CH” and “PRE_POST.INI” for each sample analysed. OpenChrom was used to produce a range of files for each sample which were saved into the sample folder. The method for producing these files using OpenChrom are described in the experimental design.

**Fig. 3:**
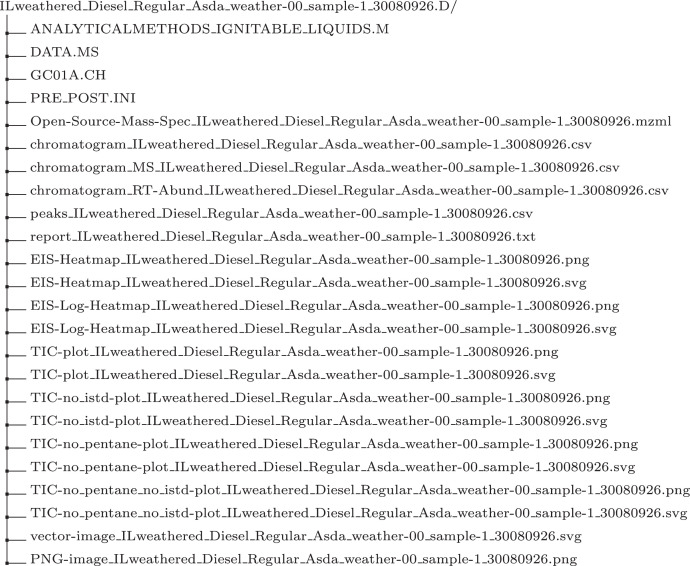
An example of a sample folder structure.

Using these conventions, the tree directory for the Diesel sample is given in Fig. 3. The description of each file according to its prefix is given below.

1. Open-Source-Mass-Spec_ILweathered_”. This file is the open-source converted format “mzML” [8] of the Agilent proprietary file “Data.MS”. A file in mzML format is readable across several open source software.
2. “chromatogram_ILweathered_”. This file contains all the information required to visualise the chromatogram and heat map. It is a table with column names “RT(milliseconds)”, “RT(minutes) - NOT USED BY IMPORT”, “RI” relative intensity and the column is an empty delimiter between RTs and m/z to intensities. It then has a variable number of columns with positive integer value names usually starting from “30” and progressed by increments of one, e.g. “31”, “32”,..., “357”. Columns “RT(milliseconds)” and “RT(minutes) - NOT USED BY IMPORT” contain retention times in milliseconds and minutes. The retention times are recorded in intervals of an average of 500 milliseconds that is exported from OpenChrom.
3. “chromatogram_MS_”. This file contains data processed from “chromatogram_ ILweathered_” to ease the production of the heat map. Only the TIS matrix with row and column headers are present. The retention time column is labelled “RetentionTimeMin”, and the m/z columns are labelled as in “chromatogram_ILweathered_”.
4. “chromatogram_RT-Abund_”. This file contains data processed from “chromatogram_ ILweathered_” to ease the production of a chromatogram. Only the TIC value pairs are present. There are two columns, the retention time and the sum of m/z intensities, titled RetentionTimeMin and Abundance respectively.
5. “peaks_”. This file consists of a table containing detected peaks, which are used to aid the determination of chemical composition. The table contains columns “RT [min]”, “Area” and “m/z” used to aid in the identification of a sample. The other columns present are automatically produced by OpenChrom and are not relevant for sample identification.
6. “reports_”. This is a general report of the sample peaks that contains meta data for the sample. It includes operator’s name (Operator) and the sample and weathering (Data Name).
7. “EIS-Heatmap_”. This prefix refers to two figures, in PNG and SVG formats, displaying a heat map produced from values in “chromatogram_MS_ILweathered_” with no transformation.
8. “EIS-Log-Heatmap_”. This prefix refers to two figures, in PNG and SVG formats, displaying a heat map produced from values in “chromatogram_MS_ILweathered_” with a log transformation. This prefix refers to the TIC plot created from the file with prefix “chromatogram_RT-Abund_”. The figure is produced in SVG and PNG formats. The figures are ready for inspection and can be used for automated analysis.
9. “TIC-no_istd-plot_”. This prefix refers to the TIC plot where the ISTD peak has been removed. The figure comes in PNG and SVG formats. ISTD peak is not informative for identification.
10. “TIC-no_pentane_”. This figure represents the TIC plot where Pentane peak removed. The removal of pentane re-scales the plot to focus on informative peaks specific to the sample analysed.
11. “TIC-no_pentane_no_istd_”. This figure represents the TIC plot where Pentane and the ISTD were removed. The figure comes in PNG and SVG formats.
12. “vector-image_ILweather_”: This figure refers to the TIC plot exported from OpenChrom. It is in SVG format with no label or axis tick values, ready for inspection. There are no axis labels to make it ready for automated image analysis.
13. “PNG-image_ILweather_”. This prefix refers to a chromatogram produced by OpenChrom and converted to PNG from SVG with file prefix “vector-image_ILweather_”. The figure is converted to PNG because it is a format that is more commonly used.

There is also a file in the root directory called “metadata” which lists all of the classifications, subclassifications, brands, weatherings, replicate numbers and Agilent file names of all the folders included in the dataset. In addition, there is a table, recorded in the file “peaks_ILweathered_metadata.csv”, consisting of data across all chromatograms. The column names are “Classification”, “Subclassification”, “Brand”, “Weather”, “Replicate”, “RetentionTime”, “Area” and “m/z”. The information of the first five columns are specific to each IL and is taken from “metadata.csv”. The last three columns are taken from the files prefixed with “peaks_”. There is also another table in the file “peaks_ILweathered_metadata_with-peak-labels.csv” containing columns named “Classification”, “Subclassification”, “Brand”, “Weather”, “Replicate”, “PeakLabel”, “RetentionTime” and “Area”. This table contains peak labels assigning the chemical compound associated with the peaks, as reported in [9]. This table content was produced using ChemStation while the table content of “peaks_ILweathered_metadata.csv” was produced with OpenChrom. This difference creates small differences in retention time which makes it somewhat complex to transfer peak labels to “peaks_ILweathered_metadata.csv”, notwithstanding this, the table enables the peaks within the OpenChrom chromatogram to be identified.

The contents of table “peaks_ILweathered_metadata_with-peak-labels.csv” is also provided as a feature table in the file “feature-table_peaks_ILweathered_metadata_with-peak-labels.csv”. The information of one sample is given in a single row. The first five columns determine the identity of the sample in the row, and they are “Classificationǥ, “Subclassificationǥ, “Brandǥ, “Weatherǥ and “Replicateǥ. The rest of the columns contain the areas of all peaks in the sample, and they are titled with an amalgamation of “PeakLabelǥ and “RetentionTimeǥ, e.g. if the peak label is “Unknownǥ and retention time is “1.15ǥ, the column titled is “Unknown_1.15ǥ. These columns are listed in increasing retention time order.

## 2 Experimental Design, Materials and Methods

The data was produced in three phases. Phase I involved data collection and preparation, phase II involved the analysis of the ILs using GCMS and phase III consisted of processing the data produced in phase II to transform it into a set of formats usable for further data analysis including machine learning applications. In phase II the Agilent ChemStation was used to perform GCMS analysis. In phase III OpenChrom [10] was used to process the Agilent proprietary data into open source formats. OpenChrom is an open source program developed to view and analyse chromatographic and other data. Specifically OpenChrom can open GCMS data acquired from most proprietary vendors and runs on macOS, Windows and Linux [1]. In addition, in Phase III, a software suite, Automated Mass spectral Deconvolution and Identification System (AMDIS), was used to identify compounds in GCMS data [11]. The program deconvolutes the data to separate individual compounds into local peaks. AMDIS is only available on Windows. To run AMDIS on Linux, users are recommended to use a Windows emulator, such as *wine* [12]. Users on Apple computers are recommended to use a virtual machine, such as VirtualBox [13], where Windows can be installed.

### 2.1 Phase I: Collection

The data was generated as part of a PhD project [9]. Petrol and diesel were collected from public fuelling stations. LPDs and MPDs were collected from local retailers in pre-sealed containers. Brands collected are listed in Fig. 1. Each IL sample was systematically evaporated and analysed by GCMS in accordance with ASTM E1618 standards [14]. A series of systematically evaporated samples were generated at 10%, 25%, 50%, 75%, 90% and 95% evaporation. Petrol samples were placed in a graduated cylinder and allowed to evaporate unaided. The LPDs, MPDs and diesel were evaporated by gently heating them in a clean round bottom flask attached to a distillation apparatus.

Prior to GCMS analysis, each IL was diluted to 2% in pentane (HPLC grade, WVR Inter- national, Leicestershire, UK) with 0.5 mg/mL tetrachloroethylene (Sigma Aldrich, >99%, St. Louis USA) as an internal standard [15].

### 2.2 Phase II: Chemical analysis

The processes in this phase were completed using Windows. Gas chromatographic analysis was performed on a Hewlett-Packard (HP) 6890/5973 gas chromatograph with a mass selective detector (GC-MSD). All data acquisitions were completed with MS ChemStation (version B.00.01 Hewlet Packard, Agilent technologies) [9]. The retention was locked to the Tetrachloroethylene peak (at RT =5.778) in order to minimise drift. A DB1-MS fused silica capillary column was used for the analysis (25.0m×0.20mmi.d.×0.33μm film thickness). The injection port was set to $250.0{\;}^{\circ }$C, the oven was set to $40.0{\;}^{\circ }$C for 5 min, then increased by $15.0{\;}^{\circ }$C every minute until $280.0{\;}^{\circ }$C was reached and then maintained for 2 min. The carrier gas was helium with a constant flow rate of 1.0 mL/min. Ion temperature source and quadrupole were set to $150{\;}^{\circ }$C and $280{\;}^{\circ }$C respectively. MS scan was in full mode (range from 30 to 300 amu) with the solvent delay set to two minutes. To carry out the injections, a 7673A Hewlett-Packard automatic liquid sampler was used. Every sample was analysed in triplicate and the injection volume for each sample was 1 µl with a 20:1 split ratio.

### 2.3 Phase III: Data engineering

The processes in this section were completed in Linux. The following steps were applied to each sample.

1. The sample folders generated by the GCMS ChemStation software were originally named with numerals, e.g. “30080926.D”. They were renamed to match their locations in the tree structure following the naming convention previously outlined, Fig. 1, e.g. ILweathered_Diesel_Regular_Asda_weather-00_sample-1_30080926.D.
2. To generate the open-source chromatogram,
  (i) right-click on the chromatogram,
  (ii) hover over “Chromatogram Export”,
  (iii) select “mzML Chromatogram (*.mzML)”,
  (iv) select the button “Use Specific Options”,
  (v) choose the exported folder location by selecting the button inline with “Export Folder” and to the right-side of the window, the button is rectangular with a single ellipsis,
  (vi) write the mzML file name in the box labelled “Filename” following the naming convention, i.e. with prefix “open-source-mass-spec_” and
  (vii) select “Finish” at the bottom right corner of the popped-up window.
3. The sample data folder produced from ChemStation can be read by OpenChrom without any processing.
4. The sample data is used in OpenChrom to detect peaks with the AMDIS database. This step requires the installation of the AMDIS database. Peak detection is achieved in OpenChrom following the steps:
  (i) right-click on the chromatogram,
  (ii) hover over “Peak Detector” and
  (iii) select “AMDIS (extern)” from the menu.
  This creates a series of windows. OpenChrom stores the peaks in memory and displays an inverted triangle in the chromatogram on top of the peaks that have been detected.
5. The peak areas were calculated using the trapezoid algorithm in OpenChrom. The areas are obtained by:
  (i) right-clicking on the chromatogram,
  (ii) hovering over “Peak Integrator”,
  (iii) selecting “Peak Integrator Trapezoid” and a window titled “Edit Processor Options” pops up,
  (iv) keeping the default button highlighted “Use System Options” and
  (v) selecting “Finish” at the bottom right corner of the popped-up window.
6. The calculated areas and retention times are stored in memory. They were saved to a comma separated value (CSV) file following the naming convention in Fig. 1, using the prefix “peaks_”. To save the detected peaks and areas,
  (i) right-click on the chromatogram,
  (ii) hover over “Peak Export”,
  (iii) select “CSV Peak Export (*.csv)”, a pop-up window titled “Edit Processor Options” will appear with radio button “Use Specific Options” already selected without modification,
  (iv) choose the exported folder location by selecting the button inline with “Export Folder” and to the right-side of the window, the button is rectangular with a single ellipsis,
  (v) next write the CSV file name in the box labelled “Filename” following the naming convention, i.e. with prefix “peaks_”.
  (vi) Select “Finish” at the bottom right of the pop-up window.
7. A report was generated in OpenChrom containing the information of the sample and the peak areas. The report was recorded in the file with prefix “reports_” in text format with extension ”*.txt”, obtaining the report by:
  (i) right-clicking on the chromatogram,
  (ii) selecting “Chromatogram Reports”,
  (iii) selecting “OpenChrom Report (*.txt)”,
  (iv) saving the report by following the same process as in item 6, replacing the prefix “peaks_” with “report_”.
8. To export the TIS as a CSV file,
  (i) right-click on the chromatogram,
  (ii) hover over “Chromatogram Export”,
  (iii) click on “CSV Chromatogram (*.csv)”
  (iv) and follow the same process as in item 6, replacing the prefix with “chromatogram_”.
9. To export the TIC as an svg file, follow the same process as item 8, click on “SVG Chromatogram (*.svg)” and replace the prefix with “vector-image_”.
10. To add the amount evaporated to the sample folder name, search for the line “Data name” inside the file prefixed with “report_”. This line contains user inputted information on the specific sample.
11. At this stage the sample file names are of the form ILweathered_Diesel_Regular_Asda_weather-00_30080926.D. where the last series of eight numbers are specific to each sample. The replicate number was added into the file name just before the last eight numbers. For example, the file name in item 1 in this list contains “sample-1” meaning that it is the first replicate.
12. The sample file prefixed with “chromatogram_” was transformed by computing a row wise summation of the m/z ion columns. The transformation yielded the abundance at each time step recorded by ChemStation. A CSV file containing only the retention time and abundance was exported to file prefixed with “chromatogram_RT-Abund_”, which is the data for creating the TIC.
13. Each sample contained an internal standard and Pentane. Three additional chromatograms were generated which removed either and both of these peaks. The peaks were removed by changing the retention time associated with each peaks to 0. The file names indicated this information with the prefixes “TIC-plot_”, “TIC-no_istd-plot_”, “TIC-no_pentane-plot_” and “TIC-no_pentane_no_istd-plot_”. Each plot was saved to in SVG and PNG formats.
14. The file generated from item 8 in this list was augmented removing extraneous columns and a new CSV file was saved prefixed with “chromatogram_MS_”.
15. The raw and log-valued TIS data was visualised using the prefixed file “chromatogram_MS_” and saved to file with the prefixes “EIS-Heatmap_” and “EIS-Log-Heatmap_” respectively, both were saved in SVG and PNG formats.

## Ethics Statement

There were no ethical requirements for the collection and analysis of the data. All software used for the curation and analysis of the dataset was open source.

## CRediT authorship contribution statement

**Jonathan Miller:** Writing – original draft, Data curation, Software. **Roberto Puch-Solis:** Writing – original draft, Supervision. **Wan Nur Syuhaila Mat Desa:** Conceptualization, Methodology, Data curation. **Niamh Nic Daeid:** Conceptualization, Methodology, Supervision, Funding acquisition, Writing – review & editing.

## Declaration of Competing Interest

The authors declare that they have no known competing financial interests or personal relationships which have, or could be perceived to have, influenced the work reported in this article.

## Data Availability

Ground Truth Data Set of UK Ignitable Liquids at varying degrees of evaporation (Original Data) (University of Dundee Discovery Portal).
